# Biosynthesized silver nanoparticles using *Polygonatum geminiflorum* efficiently control fusarium wilt disease of tomato

**DOI:** 10.3389/fbioe.2022.988607

**Published:** 2022-09-08

**Authors:** Maaz Ahmad, Ahmad Ali, Zahid Ullah, Hassan Sher, Dong-Qin Dai, Mohammad Ali, Javed Iqbal, Muhammad Zahoor, Iftikhar Ali

**Affiliations:** ^1^ Center for Yunnan Plateau Biological Resources Protection and Utilization, Yunnan Engineering Research Center of Fruit Wine, College of Biological Resource and Food Engineering, Qujing Normal University, Qujing, Yunnan, China; ^2^ Centre for Plant Sciences and Biodiversity, University of Swat, Charbagh, Swat, Pakistan; ^3^ Centre for Biotechnology and Microbiology, University of Swat, Charbagh, Swat, Pakistan; ^4^ Department of Botany, Bacha Khan University, Charsadda, Khyber Pakhtunkhwa, Pakistan; ^5^ Department of Biochemistry, University of Malakand, Chakdara, Khyber Pakhtunkhwa, Pakistan; ^6^ State Key Laboratory of Molecular Developmental Biology, Institute of Genetics and Developmental Biology, Chinese Academy of Sciences, Beijing, China

**Keywords:** silver nanoparticles, antioxidants, phenolics, flavonoids, fusarium wilt, tomato

## Abstract

Nanomaterials are gaining tremendous potential as emerging antimicrobials in the quest to find resistance-free alternatives of chemical pesticides. In this study, stable silver nanoparticles were synthesized using the aqueous extract of medicinal plant species *Polygonatum geminiflorum* **,** and their morphological features were evaluated by transmission electron microscopy, X-ray diffraction spectroscopy and energy dispersive X-ray analysis. *In vitro* Antifungal activity of the synthesized silver nanoparticles (AgNPs) and *P. geminiflorum* extract (PE) either alone or in combination (PE-AgNPs) against *Fusarium oxysporum* was evaluated using disc-diffusion and well-diffusion methods. *In planta* assay of the same treatments against *Fusarium* wilt diseases of tomato was evaluated by foliar spray method. Moreover, plant extract was evaluated for the quantitative investigation of antioxidant activity, phenolics and flavonoids by spectroscopic and HPLC techniques. Phytochemical analysis indicated the presence of total phenolic and flavonoid contents as 48.32 mg ± 1.54 mg GAE/g and 57.08 mg ± 1.36 mg QE/g, respectively. The DPPH radical scavenging of leaf extract was found to be 88.23% ± 0.87%. Besides, the HPLC phenolic profile showed the presence of 15 bioactive phenolic compounds. Characterization of nanoparticles revealed the size ranging from 8 nm to 34 nm with average crystallite size of 27 nm. The FTIR analysis revealed important functional groups that were responsible for the reduction and stabilization of AgNPs. In the *in vitro* assays, 100 μg/ml of AgNPs and AgNPs-PE strongly inhibited *Fusarium oxysporum*. The same treatments tested against *Fusarium* sprayed on tomato plants in controlled environment exhibited nearly 100% plant survival with no observable phytotoxicity. These finding provide a simple baseline to control *Fusarium* wilt using silver nano bio-control agents without affecting the crop health.

## Introduction

All over the world, agricultural crops are severely affected by phytopathogenic microbes which cause a variety of diseases and lead to reduction in their overall productivity (Savary et al., 2012; Rafique et al., 2017; Saratale et al., 2018). Disruption of the productivity of important crops due to pathogenic diseases is a serious threat to the global food security (Savary et al., 2012). Different fungi, bacteria and viruses produce toxic substances that are harmful to the health of beneficial life forms, thereby compromising the quality and quantity of crops (Bashir et al., 2018; Gao et al., 2018). Conventionally, synthetic chemicals have been used to control these pathogenic diseases; however, their frequent administration is hazardous for non-targeted organisms (Gardener and Fravel, 2002; Hajek and Eilenberg, 2018). Therefore, the introduction of novel and targeted treatments is needed as a safer and alternative strategy to control plant pathogens (Leon-Buitimea et al., 2020; Avila-Quezada et al., 2021).

The field of nanotechnology, ensuing from the coupling of material science and biotechnology, is focused to develop novel agents for the effective control of phytopathogens (Zhang and Webster, 2009; Saxena et al., 2012). Nanoparticles have tremendous importance due to their various applications in the fields like medicine, biology, chemistry, material science and physics (Albrecht et al., 2006; Thiruvengadam et al., 2006). Chemical and physical methods used for the synthesis of nanoparticles are costly and produce substances that are toxic to the health of living organisms (Yaqoob et al., 202). In contrast, biological synthesis is a simple and viable method that utilizes plants, microorganisms, polysaccharides and enzymes as synthesis substrates (Hebbalalu et al., 2013; Khan et al., 2022).

As nano factories, green plants provide relatively modest, environment-friendly and a faster way for nanoparticles synthesis on large scale (Abou El-Nour et al., 2010). Plant secondary products act as reducing and stabilizing agents to transform metal salts into stable nanoparticles (Kuppusamy et al., 2016). At low concentrations, green synthesized silver nanoparticles have documented antimicrobial activities against plant and human pathogens with no cytotoxicity (Jeong et al., 2005; Sharma et al., 2009; Alam et al., 2018).

*Polygonatum Mill* is a genus of rhizomatous, perennial, monocot herbs in the family Asparagaceae. The genus is represented by about 75 species in the world, distributed primarily in the temperate and alpine regions of the northern hemisphere, extending into mountains in the subtropical region as well (Chase et al., 2016; Floden and Schilling, 2018). *Polygonatum* species are characterized by sympodial rhizomes, and opposite to verticillate leaves and fleshy berries (Wang et al., 2022). The subterranean rhizome in many species of the genus have been utilized in traditional as well as modern medicine for the cure of several diseases. *Polygonatum* species are used as antidiabetic, as coolant, immunostimulatory, to treat respiratory problems (Jiao et al., 2018). Some species (*P. cyrtonema, P. sibiricum*) are also listed in Chinese Pharmacopoea. Recently it has been found that some species of *Polygonatum* are rich in proteins and nutrients and can become potential future grain crop (Si and Zhu, 2021. The genus *Polygonatum* includes important medicinal plants which are utilized for treating several human ailments such as diabetes, vertigo, ringworm and pulmonary problems, and exhibit hepatoprotective and antioxidant activities (Son et al., 1990; Zhao et al., 2018). Moreover, the wide use of *Polygonatum* species in various traditional systems of medicines as antidiabetic, aphrodisiac, antituberculant, tonic, diuretic etc., has previously been reported (Khan et al., 2013; Suyal et al., 2020; Sharma et al., 2021).

*P. geminiflorum Decne*. locally known as “Peramole” (Pashto), is an important rhizomatous perennial herb inhabiting temperate coniferous forests, and alpine zone in northern Pakistan’s Western Himalayan and Hindukush Mountain region, extending westward to Afghanistan (Chase et al., 2016)*.* This species is closely related to P. verticillatum and is known to people by the same vernacular names “Noore-Alam and Peramole”. The rhizome of *P. geminiflorum* as well as *P. verticillatum* is crushed and fried in wheat flour and given to lactating mothers for increasing milk (Rahman et al., 2022)*.* Khan and Khatoon, (2008) have reported that the local people in northern Pakistan use *P. geminiflorum* for the treatment of uterine tumor, menstrual abnormalities, gout, and skin diseases. Moreover, Khan and Rauf, (2015) have comprehensively described the phytochemical constituents of different species in the genus, and the associated antimibacterial, antifungal and antioxidant activities. This study has also reported that *P. geminiflorum* is rich in phenolics, saponins, alkaloides and phytoharmones, and therefore is a high value medicinal plant, like the most commonly explored *P. vericillatum.* Till date, no published report is available on the synthesis of AgNPs using *P. geminiflorum*. Therefore, the present study was aimed to synthesize biologically stable silver nanoparticles using aqueous extract from *P. geminiflorum* and to test the synthesized nanoparticles against Fusarium wilt disease of tomato *in vitro* and in planta.

## Materials and methods

### Extract preparation for phytochemical analysis

For phytochemical analysis, methanolic extract was prepared by dissolving 50 g shade-dried powdered leaves of *P. geminiflorum* in 100 ml methanol (95%), followed by incubation at 28°C for 24 h. After incubation the solution was filtered, evaporated and 15 g final mass of crude extract was obtained which was then used for different phytochemicals tests.

### Phenolics, flavonoids and DPPH antioxidant activity

TPC was determined according to the Folin-Ciocalteu’s colorimetric method as discussed by Singleton and Rossi, (1965) using calibration curve of standard gallic acid. Briefly, 5 mg of crude extract was added to dH_2_O (10 ml), and the solution was heated in a water bath for 30 min and then filtered into a vial. About 600 µl of the filtrate was taken and mixed with 100 µl of Folin-Ciocalteu’s reagent, followed by addition of Na_2_CO_3_ (sodium carbonate, 7%). The mixture was kept at room temperature for 90 min in dark. After the reaction mixture turned blue, the absorbance was recorded at 760 nm.

TFC determination was carried out by Aluminum chloride (AlCl_3)_ colorimetric method as described in Chang et al. (2002) using standard quercetin curve. Approximately 5 mg of crude extract was added to dH_2_O (10 ml), the solution was heated in a water bath for 30 min, filtered and five different dilutions (62.5 μg/ml, 125 μg/ml, 250 μg/ml, 500 μg/ml and 1,000 μg/ml) were prepared. From each dilution, 100 µl of extract was thoroughly mixed with 500 µl dH_2_O and 100 µl of NaNO_3_ (5%), 150 µl of AlCl_3_ (10%) and 200 µl of sodium hydroxide (1 M). The absorbance of reaction mixtures was recorded at 510 nm by using UV-VIS double beam spectrophotometer.

The antioxidant assay was determined as DPPH radical scavenging by following the method of Goyal et al. (2010) using standard ascorbic acid.

### HPLC–UV analysis for determination of phenolics

Determination of the phenolics in leaf extract was accomplished using HPLC Agilent 1,260 system equipped with UV detector, following Zeb, (2015) with minor modifications. Approximately, 1 g dried sample was mixed with 50% methanol (v/v; 20 ml) and the solution was placed in hot water bath at 50^°^C for 1 h. The solution was filtered 2 times and poured into HPLC vials for the detection of phenolic compounds. For the separation of components, ZORBAX Eclipse C18 (4.6 mm × 250 mm, 5 Micron) column was used and the identification of compounds was carried out by the comparison of retention times with available standards and those reported in literature.

### Biosynthesis of silver nanoparticles

The biosynthesis of silver nanoparticles from *P. geminiflorum* leaf extract was carried out using the protocols of Ali et al. (2016). Briefly, plant aqueous leaf extract (20 mg/ml) was prepared by heating 2 g shade-dried and powdered leaves of *P. geminiflorum* in 100 ml distilled water until boiled. The prepared extract was cooled down at room temperature and filtered three times using Whatman No. 1 filter paper. Distilled water was added to adjust the final volume as 100 ml. The stock extract was then diluted to 5 mg/ml by adding distilled water. The extract was stored at 4°C until used for nanoparticles synthesis.

For silver nanoparticles synthesis, 5 ml of the plant aqueous extract (5 mg/ml) was mixed with 5 ml of AgNO_3_ (4 mM) solution in a test tube. The reaction mixture was exposed to sunlight for 15 min and the color change was monitored following the reaction of both reactants. The mixture was incubated at room temperature and spectral readings were recorded at various time points until consistency in surface plasmon resonance was achieved after 48 h. The synthesized AgNPs were purified by following the protocol of Arif et al. (2022). Briefly, the samples were centrifuged at 15,000 rpm, the supernatants were discarded, and the pellets were dissolved in deionized distilled water by using ultrasonic sonicator. The process of centrifugation and washing was repeated three times until purified AgNPs were obtained. The purified nanoparticles were kept at room temperature for drying which were later subjected to various characterization techniques. To prepare plant extract-encapsulated silver nanoparticles (AgNPs-PE), appropriate volumes of 5 mg/L plant extract was added to dried AgNPs in falcon tubes (15 ml).

### Characterization of AgNPs

Characterization of the synthesized AgNPs was accomplished using different physical techniques, as has been previously reported (Gopinath and Velusamy, 2013; Ali et al., 2016).

The UV-visible spectral analysis was recorded to find out the characteristic peak for silver nanoparticles. The spectral range of 300 nm–600 nm was used to monitor the surface plasmon resonance. For this purpose, the Multiskan™ Sky Microplate Spectrophotometer (MAN0018930) was used.

The FTIR characterization were performed using Thermo-Nicolet 6,700 FTIR Spectrometer (Madison, WI, United States) with spectrum ranging from 4,000 cm^−1^ to 400 cm^−1^. The functional groups were detected using Ge crystal in ATR reflection mode. The functional groups responsible for the formation of stable nanoparticles were identified by comparing observed FTIR peaks with IR spectrum table.

The TEM analysis of the biosynthesized AgNPs were performed on JEOL JEM-101 system. Different magnification lens was employed for exploration of the shape, size and morphology of prepared AgNPs. Further, SAED (selected area electron diffraction) determined the crystalline nature of AgNPs.

For EDX study, scanning electron microscope (JSM5910, JEOL, Japan) equipped with energy dispersive x-ray system was used. The EDX characterization determined the elemental composition for the synthesized silver nanoparticles.

The XRD analysis of biosynthesized AgNPs were performed through JDX-3432, JEOL, Japan. The average crystallite size for the prepared nanoparticles were calculated using Debye–Scherrer equation which is assessed by:

D=Kλ/β.cosθ

## Antimicrobial activities

### *In vitro* inhibition assay of *Fusarium oxysporum*

The *in vitro* antifungal activity of AgNPs was performed against *F. oxysporum* using well-diffusion and disc-diffusion methods following Gopinath and Velusamy, (2013), with certain modifications. Briefly, the confirmed fungal strain *F. oxysporum* was taken from the plant pathogens facility at the Centre for Plant Sciences and Biodiversity, University of Swat, Pakistan. The strain was cultured and maintained on PDA plates. For well diffusion, four wells of equal size were made on PDA plates and each well was loaded with 100 µl of either AgNPs (100 μg/ml), plant extract (5 mg/ml) or AgNPs-PE (100 μg/ml). The same volume of distilled water was taken as control. The cultures were incubated at 28°C and the zone of inhibition (mm) around the wells was recorded after 4 days of culture. Similarly, disc-diffusion method was carried out by employing the same treatments and concentrations as for well-diffusion method. The *F. oxysporum* was cultured and four discs (each poured with 20 µl solution) were placed on PDA plate. The inhibition zone was measured after incubation at 28°C for 4 days (Table 1).

**TABLE 1:** Zone of inhibition (in millimeter) of *F. oxysporum* in disc and well diffusion method.

Zone of inhibition in millimeter (mm)	Method	Ag NPs (100 μg/ml)	Plant extract (5 mg/ml)	Plant coated Ag NPs (100 μg/ml)	Control
	Well diffusion	11 ± 1	5 ± 1	18 ± 1	No Inhibition
	Disc diffusion	9 ± 1	3 ± 1	14 ± 1	No Inhibition

### *In planta Fusarium oxysporum* inhibition assay

The *in planta* experiment against *F. oxysporum* was performed following the protocol of Ali et al. (2015) with some modifications. Briefly, the culture of *F. ozysporum* were grown overnight in potato dextrose broth (PDB) and a final concentration of 1.5 × 10^4^ conidia ml^−1^ was adjusted. Further, the seeds of *Solanum lycopersicon* were sown in a greenhouse upheld at 24°C ± 5°C and a photoperiod of 14-h day/10-h night. The 18 days old seedlings were transferred to pots and kept under the same temperature and photoperiod. After 10 days, the plants were sprayed until excess with AgNPs (100 μg/ml), plant extract (5 mg/ml) and AgNPs-PE (100 μg/ml). Commercial fungicide (bromuconazole 100 μg/ml) was taken as positive control and water as negative control. Each treatment was replicated three times and each replication was consisted of nine plants placed in plastic pots. After 24 h, each treated plant was drenched with 30 ml of *F. oxysporum* (concentration of 1.5 × 10^4^ conidia ml^−1^) PDB culture. After inoculation the treated plants were observed for one week and the disease spontaneity or inhibition was recorded in terms of percent plant survival. The obtained data was statistically analyzed using student’s t test to find out the significance of difference between the treatments for the percent healthy plants.

## Results

### Phytochemical analysis

**FIGURE 1:**
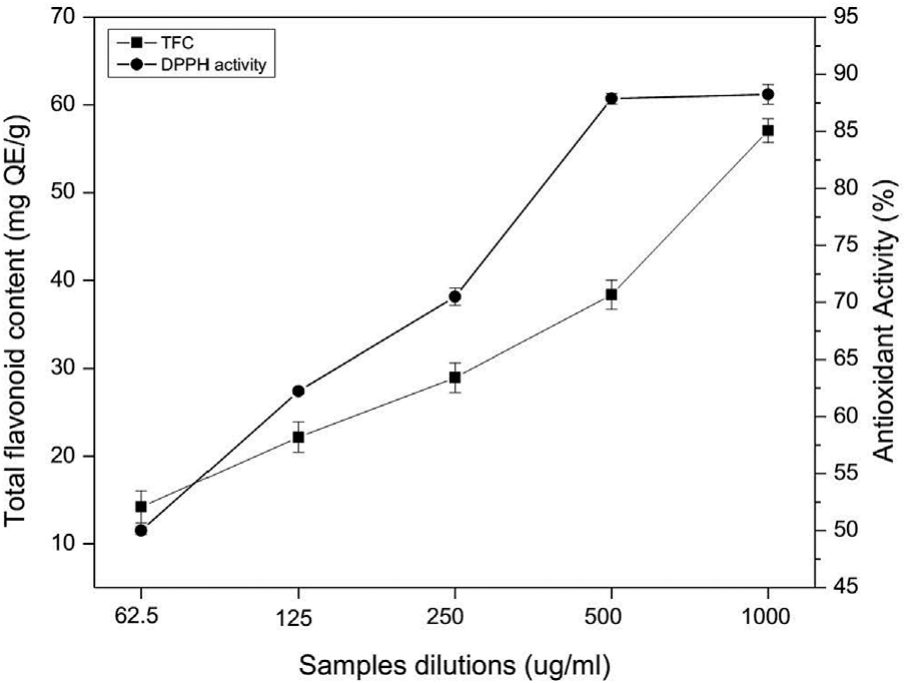
Total flavonoid content and DPPH free radical scavenging capability of *P. geminiflorum*.

**FIGURE 2:**
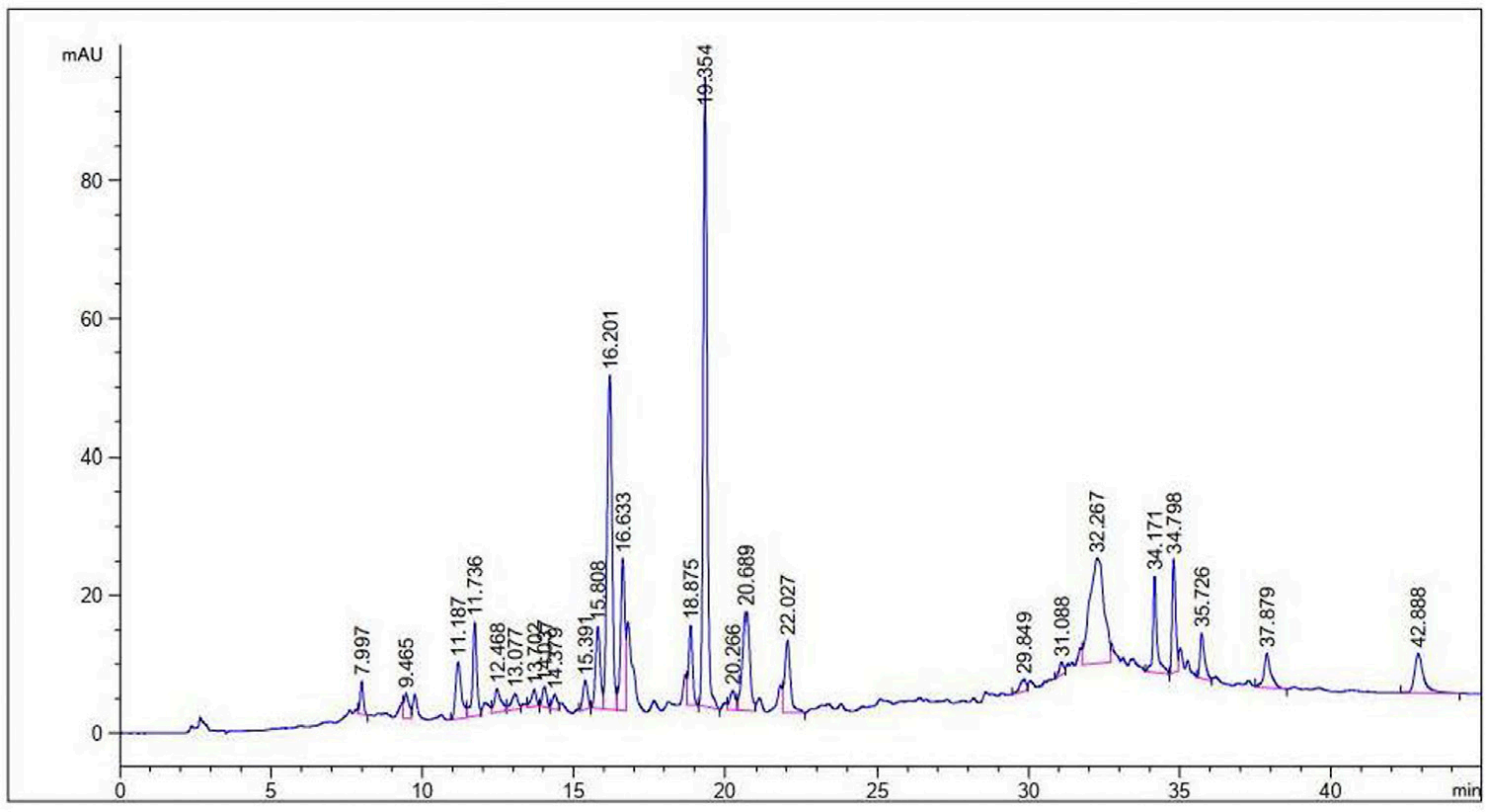
HPLC chromatogram representing phenolic profile of the leaf extract of *P. geminiflorum*.

Total phenolic content of the aqueous and methanolic extract of *P. geminiflorum* is given in Table 2, which was estimated using the regression equation of standard gallic acid. The TPC for both extracts were found 42.27 mg ± 1.73 mg and 48.29 mg ± 1.54 mg GAE/g, respectively. Regarding, total flavonoid contents, the highest TFC was found in the dilution of 1,000 μg/ml that is presented in Figure 1. Further, results regarding DPPH free radical scavenging capability of *P. geminiflorum* methanolic leaf extract revealed the highest scavenging percent for the concentration of 1,000 μg/ml. The obtained results were compared with standard ascorbic acid and the data of DPPH assay is presented in Figure 1. The HPLC analysis revealed fifteen bioactive phenolic compounds in the leaf extract of *P. geminiflorum* (Figure 2). The most prominent possible compounds identified were P-coumaric acid derivative, ellagic acid, p-hydroxy benzoic acid, Vanillic acid, rutin and quercetin-3-malonylglucoside (Figure 2; Table 3).

**TABLE 2:** The total phenolic content in *P. geminiflorum* methanolic and leaf extract.

S. No.	Extract sample	TPC (mg GAE/g). Mean ± S.E.M
1	Aqueous	42.27 ± 1.73
2	Methanolic	48.29 ± 1.54

**TABLE 3:** Phenolic compounds identified in HPLC profiling of *P. geminiflorum* leaf extract.

Peak area (mAU*s)	Retention time (min)	Width (min)	Height (mAU)	Peak area (%)	Proposed identity of compound	Identification reference
18.227	31.088	–	–	–	Mandelic acid	Standard
24.333	29.849	–	–	–	Caffeic acid	Standard
34.914	13.077	0.233	2.224	0.907	Isovitexin-4-o-glucoside	Ovais et al. (2018)
36.403	20.266	–	–	–	Catechin hydrate	Standard
43.033	9.465	–	–	–	Quercetin	Standard
52.137	12.468	–	–	–	Morin	Standard
70.043	35.726	0.156	6.526	1.819	Kaempferol-3-O-sophorotrioside-7-glucoside	Litchfield and Wilcoxon (1949)
105.330	18.875	0.138	11.560	2.736	Apigenin-7-o-rutinoside	Ovais et al. (2018)
128.141	15.808	0.166	11.796	3.323	Hydroxy benzoic acid derivative	Ovais et al. (2018)
130.796	34.171	0.134	14.021	3.397	Quercetin-3-malonylglucoside	Litchfield and Wilcoxon (1949)
138.683	22.027	–	–	–	Rutin	Standard
203.439	20.689	0.212	14.378	5.283	Vanillic acid	Ovais et al. (2018)
221.364	16.633	0.150	21.953	5.749	p-hydroxy benzoic acid	Ovais et al. (2018)
568.275	16.201	–	–	–	Ellagic acid	Standard
828.884	19.354	0.141	91.170	21.527	P-coumaric acid derivative	Ovais et al. (2018)

### Characterization of the synthesized AgNPs

**FIGURE 3:**
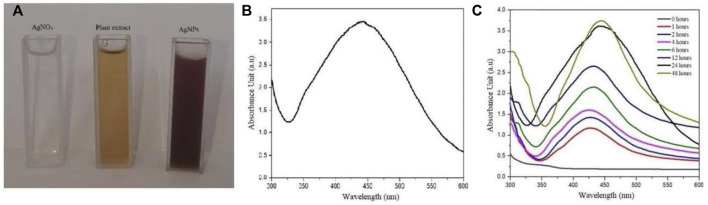
**(A)** Color change of reaction mixture, **(B)** and **(C)** UV-Visible absorbance spectrum of the biosynthesized AgNPs.

The reaction mixture was turned brown after mixing the equal volume of AgNO_3_ (4 mM) with plant extract (5 mg/ml) at room temperature. Plant extract reduced the AgNO_3_ solution to Ag ions and capped the Ag^+^ with important secondary constituents. The silver ions in the presence of plant secondary constituents were stabilized into Ag nanoparticles. The appearance of brown color was due to silver ions reduction which is a general characteristic for the AgNPs biosynthesis. No color change was observed after 24 h (Figure 3A).

The UV-Vis spectral analysis showed an increase spectrum between 380 nm and 500 nm where the highest absorbance peak was recorded at 440 nm. Moreover, the solution was observed under UV-Vis spectra at different time interval for 30 days showing no momentous change in the absorbance spectrum after 48 h (Figures 3B,C).

**FIGURE 4:**
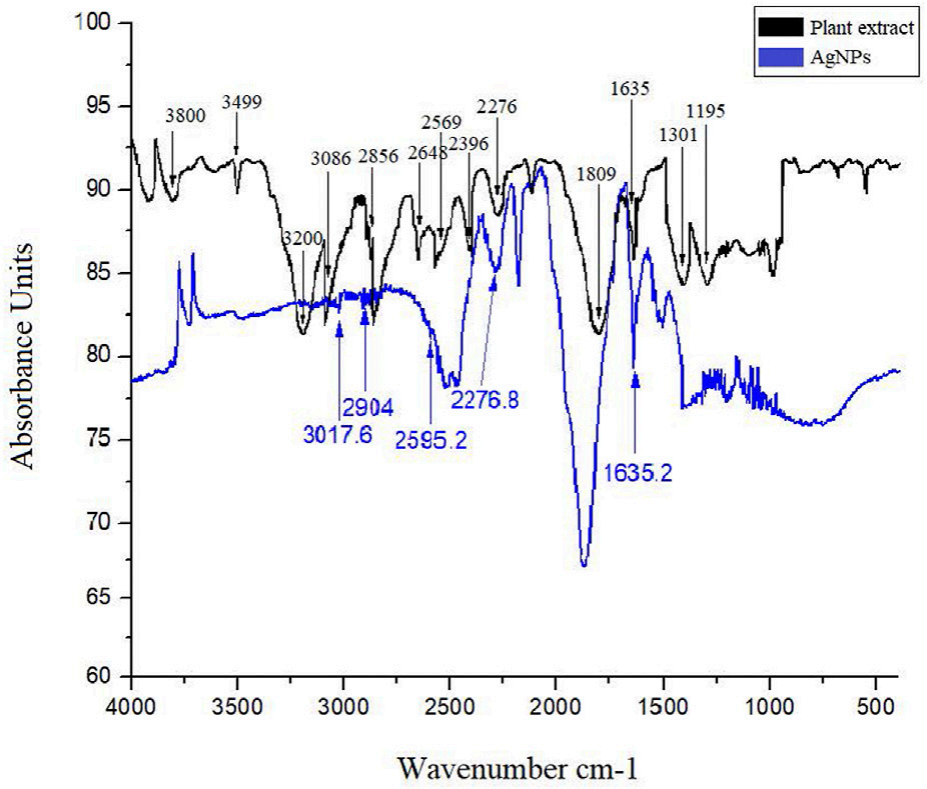
FTIR spectrum of plant extract and AgNPs showing bands for various functional groups responsible for stabilization of silver nanoparticles.

FTIR analysis of the plant extract identify different peaks at specific wavenumber indicating the occurrence of various functional groups. Briefly, broad peaks at 3,499 cm^−1^ and 3,200 cm^−1^ was due to the O-H stretching of alcohol. A medium peak at 3,086 cm^−1^ was found due to the C-H stretching of alkene. A weak band at 2,569 cm^−1^ was detected for the S-H stretching of thiol. At 2,276 cm^−1^ a strong broad band was observed for the N = C = O stretching of isocyanate. A strong peak at 1809 cm^−1^ was due to the C = O stretching of acid halide. A medium peak at 1,635 was present due the C = C stretching of alkene. Similarly, the FTIR analysis of AgNPs also revealed various peaks for specific functional groups. Concisely, a weak broad peak at 3,017.6 cm^−1^ and 2,904 cm^−1^ was detected for the O-H stretching of alcohol. A weak peak at 2,595.2 cm^−1^ was observed for the S-H stretching of thiol and at 2,276.8 cm^−1^ a strong broad band was detected due to N = C = O stretching of isocyanate. A medium band at 1,635.2 cm^−1^ was observed due to C = C stretching of conjugated alkene. These groups were probably detected due to the capping layers of the plant secondary metabolites and resulted with stable formation of AgNPs (Figure 4).

**FIGURE 5:**
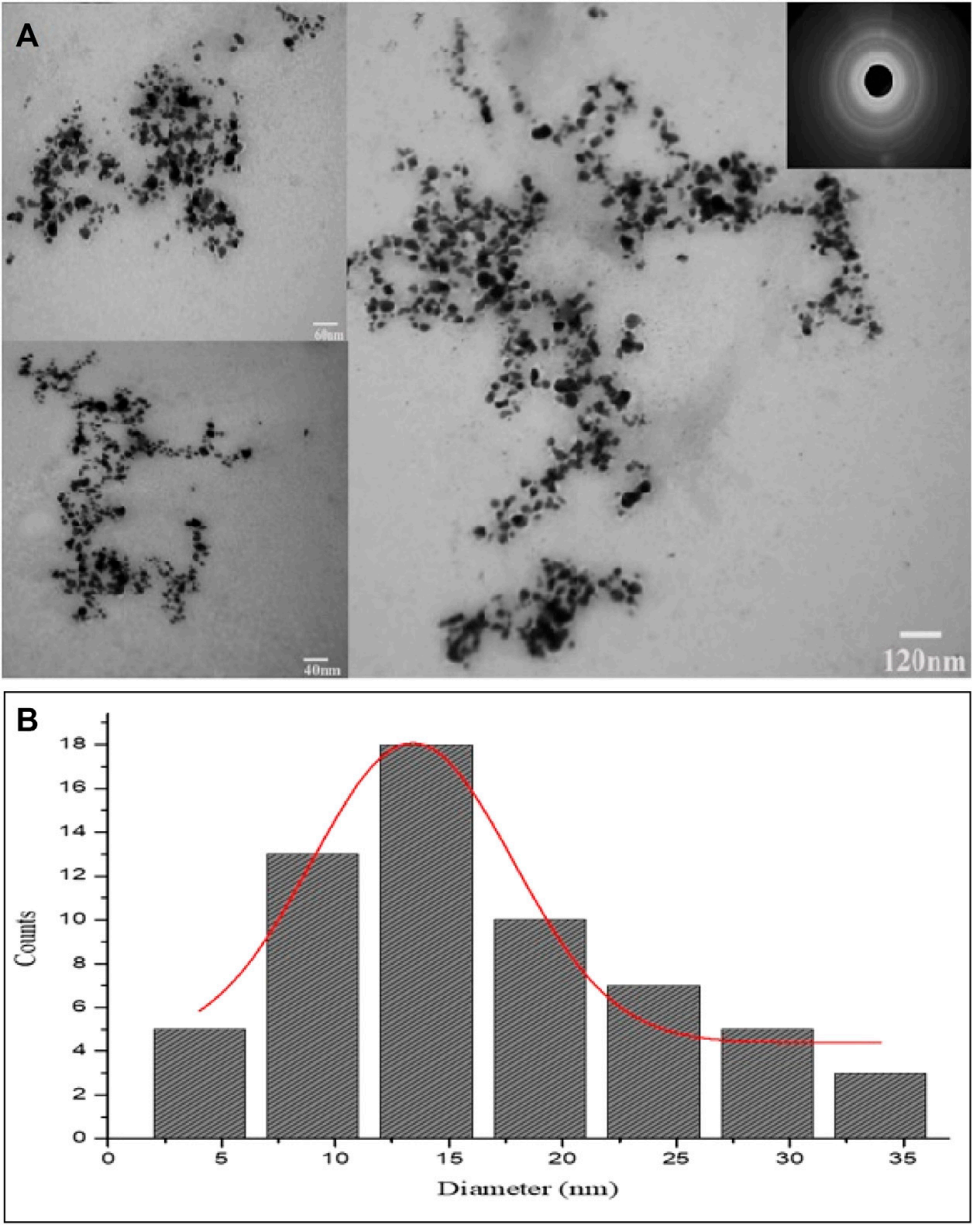
**(A)** TEM images and SAED pattern of the green synthesized AgNPs. **(B)** Histogram showing size distribution of the synthesized AgNPs.

The size and shape details of the biosynthesized AgNPs were analyzed with TEM. The TEM micrographs showed different shapes for AgNPs but maximum particles were round or spherical in shape. It can be clearly seen in TEM images that the size of the biosynthesized AgNPs ranged from 8 nm to 34 nm and most of them were monodispersed. The TEM observations showed that due to presence of capping agents the prepared AgNPs were not in straight contact even inside aggregates. Further, Brags reflections rings were recorded in selected area electron diffraction (SAED) study that were corresponding to the crystalline nature of AgNPs (Figure 5A). Histogram showing size distribution of the synthesized AgNPs is shown in Figure 5B.

**FIGURE 6:**
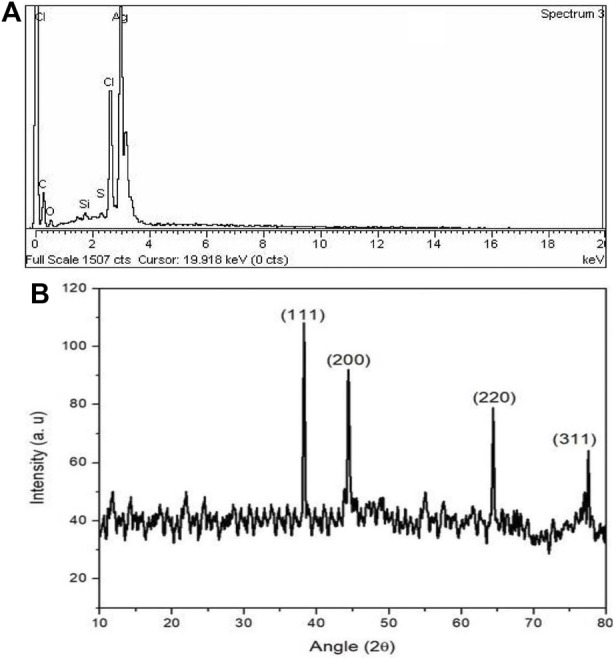
**(A)** Energy dispersive x-ray and **(B)** XRD pattern of the biosynthesized AgNPs.

The elemental composition for the prepared AgNPs were determined using energy dispersive x-ray spectroscopy. The major peaks for Ag, Cl, C, O and S elements were found at EDX spectra having the weight percentage 77.85, 15.44, 4.92, 1.50 and 0.30 respectively. The high energy peak spectrum between 3 KeV and 4 KeV was noticed which is particular for Ag element (Figure 6A). Similarly, the XRD study were performed over 2*θ* diffraction angle ranging from 10° to 80° that showed four different Bragg’s reflections. The XRD diffraction peaks AgNPs were located at 38.30, 44.35, 64.40, and 77.55 equivalent to the silver crystal planes of (111), (200), (220), and (311) respectively. The average crystallite size of AgNPs found according to Debye–Scherrer equation was 27 nm (Figure 6B).

### *In vitro* inhibition of *F. oxysporum*

**FIGURE 7:**
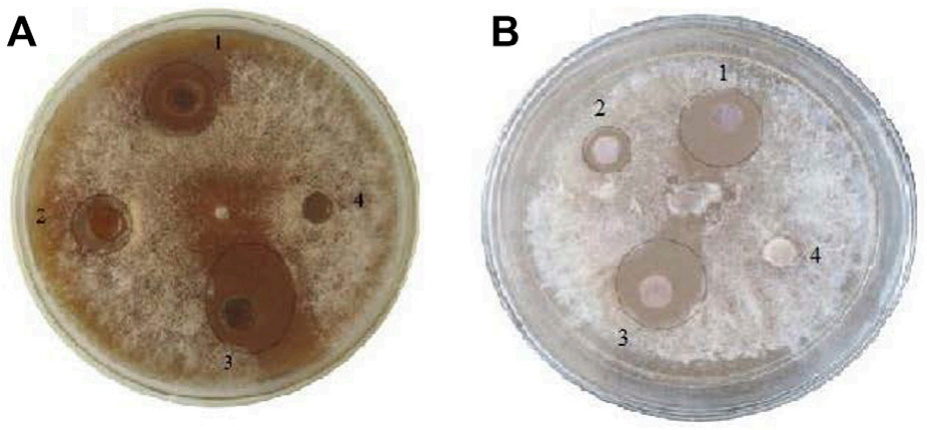
*In vitro* well diffusion. **(A)** and disc diffusion, **(B)** methods. AgNPs treated, 1 plant extract treated, 2 AgNPs-PE combined treated, 3 and water as control 4.

The AgNPs (100 μg/ml), plant extract (5 mg/ml) and AgNPs-PE (100 μg/ml) strongly inhibited the growth of *F. oxysporum* in the well and disc diffusion methods. The AgNPs-PE exhibited highest inhibition, followed by AgNPs and plant extract while no inhibition in response to water (control) was observed. In well diffusion method, the AgNPs-PE, AgNPs and plant extract inhibited the growth by 18 mm ± 1 mm, 11 mm ± 1 mm and 5 mm ± 1 mm respectively while in disc diffusion method it was 14 mm ± 1 mm, 9 mm ± 1 mm and 3 mm ± 1 mm respectively (Figure 7).

### *In planta* inhibition of *F. oxysporum*

**FIGURE 8:**
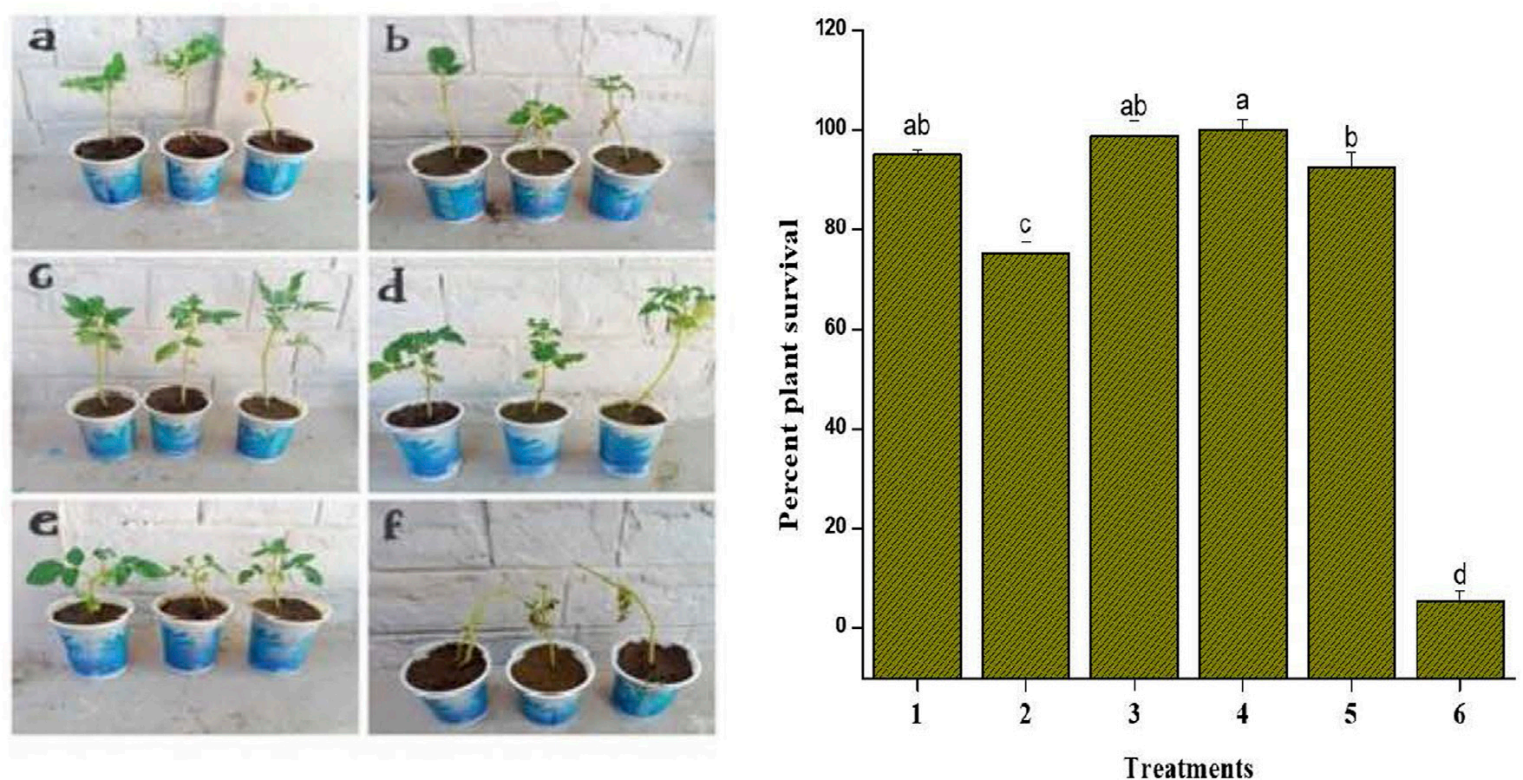
In planta treatments against F. oxysporum. Bar graph showing plant survival percent after the inoculation of F. oxysporum. Treatments included: 1 AgNPs (100 μg/ml); 2 plant extract (5 mg/ml); 3 AgNPs-PE (AgNPs 100 μg/ml + PE 5 mg/ml); 4 fungicide (bromuconazole 100 μg/ml); 5 AgNPs (50 μg/ml); 6 Control. Different letters are representing statistically significant differences after performing Tukey HSD test.

To investigate the *in planta* antifungal activities against the *Fusarium* wilt of tomato, the pot grown plants were treated with AgNPs (100 μg/ml), plant extract (5 mg/ml), AgNPs-PE (100 μg/ml), fungicide bromuconazole (100 μg/ml) as positive control and water as negative control. After inoculation, the control plants treated with water started yellowing of the leaves, followed by wilting and stunting of growth. However, the plants treated with fungicide inhibited the pathogen and the plants showed optimum growth. Similarly, no wilting symptoms were observed in response to both concentrations (100 µg) of AgNPs. However, the plants treated with plant extract showed partial onset of wilting symptoms. Overall, the AgNPs-PE completely inhibited the *F. oxysporum* growth and the plants were healthy and showed maximum growth. The percent plant survival after inoculation of *F. oxysporum* in response to different treatment is shown in Figure 8. The obtained results of *in planta* experiment were correlated with the results of *in vitro* experiment and both showed comparable effects. Moreover, the effects of different treatments on root and shoot length, and on biomass has been presented in Table 4.

**TABLE 4:** Effect of different treatments on the growth parameters of tomato during in planta experiment against F. oxysporum. Different letters are showing statistical differences among treatments at *p* < 0.05 after performing Tukey HSD test.

Treatment	Fresh biomass (g)	Root length (cm)	Shoot length (cm)
AgNPs (100 μg/ml)	26.98^b^	11.22^b^	20.22^b^
Plant extract (5 mg/ml)	20.72^d^	5.16^d^	15.16^d^
AgNPs-PE (AgNPs 100 μg/ml + PE 5 mg/ml)	28.93^a^	12.56^a^	22.56^a^
Fungicide (bromuconazole 100 μg/ml)	29.34^a^	12.78^a^	22.78^a^
AgNPs (50 μg/ml)	23.12^c^	10.12^c^	18.12^c^
Control	13.43^e^	2.60^e^	11.61^e^

## Discussion

The overuse of synthetic pesticides leads to disease resistance in microbes which is becoming a major hazard to health of beneficial life forms including human (Rudramurthy et al., 2016). These resistant microbes cause various diseases and reduce the yield and quality of crops (Fletcher et al., 2006). An example of such diseases is *Fusarium* wilt of tomato which negatively affect the plant growth and productivity (Prajapat et al., 2013; Özkara et al., 2016. However, due to their non-hazardous nature, silver nanoparticles have been used as antimicrobial agents against disease causing microbes (Burdusel et al., 2018; Liao et al., 2019). Several reports have shown the effectiveness of green synthesized AgNPs against a variety of microbes due to the combined effects of plant secondary metabolites and Ag metal (Kim et al., 2007; Choudhury et al., 2016; Durán et al., 2016; Marslin et al., 2018). Moreover, plant based synthesis of silver nanoparticles is economically feasible, efficient against pathogens and non-toxic (Liaqat et al., 2022; Ahmed and Mustafa, 2020).

In the present report, *P. geminiflorum* leaf extract was investigated for phytochemicals and antioxidant activity along with antifungal properties of the biosynthesized AgNPs. Plant secondary metabolites including terpenoids, flavonoids, phenolics etc. may act as reducing and stabilizing agents in green synthesizing nanoparticles; however, complexity about the exact mechanism of synthesis exists (Marslin et al., 2018). This may be attributed to the synergism of these biomolecules in reduction of metal ions and variable phytochemical profiles of different plant extracts leading to uncertainty about a generalized mechanism. The potential role of these biomolecules in synthesis of nanoparticles has been extensively discussed by Mustapha et al. (2022) and Siddiqi et al. (2018). Jain and Mehata, (2017) and Pradeep et al. (2021) have recently documented reduction of silver ions through standard phenolic compounds. Still, other metabolites like terpenes have also been documented to exhibit dual role i.e. capping as well as reducing agents (Mashwani et al., 2016). TPC in methanolic extract (48.32 mg ± 1.54 mg GAE/g) and aqueous extract (42.30 mg ± 1.73 mg GAE/g) was found to be comparable. Moreover, the highest total flavonoid content found in 1,000 μg/ml was 57.08 mg ± 1.36 mg QE/g which was compared with previous studies (Gupta et al., 2018; Nazir et al., 2020). Highest DPPH radical scavenging activity of 88.23% ± 0.87% was exhibited by the methanolic extract. Moreover, a total of fifteen phenolic compounds were found in the leaf extract with respect to standards and reported data. The results were compared with previous studies on HPLC based phytochemical investigations of *Pistacia integerrima*, *Pisum sativum* L. *Ziziphus oxyphylla, Grewia optiva* (Nazir et al., 2020; Sharifi-Rad et al., 2020). Results depicted rich phytochemical profile and significant antioxidant properties of the investigated plant extract which suggest its probable involvement as the major reducing compounds of silver ions. The solution turned brown due to reduction of silver ions in reaction mixture which indicated the formation of silver nanoparticles, and the result was compared with previously reported studies on green nanoparticles synthesis (Ali et al., 2016; Arif et al., 2022). Moreover, a characteristic surface plasmon resonance peak at 440 nm during UV-Vis analysis was observed which further confirmed the synthesis of nanoparticles. The obtained UV-Vis spectral data was compared with previous studies which showed similar pattern for silver nanoparticles (Ali et al., 2015; Marslin et al., 2018; Ahmed and Mustafa, 2020).

The FTIR spectra identified various functional groups responsible for the synthesis process of AgNPs. These functional groups were likely responsible for synthesis of and providing capping layers to Ag nanoparticles. Therefore, the synthesized AgNPs were more stable and non-toxic as previously been discovered by Arif et al. (2022). The TEM analysis revealed spherical or round shaped monodispersed AgNPs that were not in direct contact because of the capping layers of secondary metabolites. The SAED analysis showed the synthesized particles as of crystalline nature. The EDX study showed an intense peak of Ag metal with a weight of 77.85%. The TEM and EDX based results about the physical characteristics of nanoparticles were positively correlated with previous studies (Khan et al., 2020).

The XRD analysis revealed the planes of 111, 200, 220, and 311 at diffraction peaks of 38.30, 44.35, 64.40, and 77.55, respectively. Moreover, the Debye–Scherrer equation showed the average size of 27 nm and the obtained results were compared to the previous literature studies (Bindhu et al., 2020; Ghojavand et al., 2020).

The prepared AgNPs showed substantial antifungal activity against *F. oxysporum*. The *in vitro* experiment resulted in significant inhibition potency of AgNPs-PE against the tested fungus. The plant extract has important secondary constituents that coat and increase the antimicrobial effects of Ag metal. Further, antifungal activities of the AgNPs-PE, AgNPs (100 μg/ml), plant extract and fungicide were compared using *in planta* experiment. The *in planta* experiment revealed efficiency of the AgNPs-PE with the highest percent survival of plants. However, the AgNPs (50 μg/ml) showed pronounced antifungal activity because no symptoms of leaf yellowing (chlorosis) was observed throughout the experiment. The obtained results of *in vitro* and *in planta* experiments are in general agreement and much promising when correlated with the previously reported studies on antimicrobial activities of silver nanoparticles (Jo et al., 2009; Ali et al., 2015; Gopinath et al., 2017; Some et al., 2018; Alam et al., 2019; Haroon et al., 2019; Santiago et al., 2019; Vanti et al., 2019; Renuka et al., 2020; Rizwan et al., 2020; Tariq et al., 2021). The green AgNPs in the presence of plant capping agents did not allow particles to aggregate and increases its long-term stability (Spagnoletti et al., 2021). The plant extract carries out dual function i.e., perform the reduction of silver ion and stabilization of AgNPs in the reaction mixture (Sunkar and Nachiyar, 2012). The AgNPs-PE showed the highest activity because of its increased antifungal property due to the presence of plant secondary chemicals.

Green synthesized nanoparticles are considered advantageous because of its non-toxicity, environment friendly nature, are more economical and sustainable (Alsammarraie et al., 2018; Ying et al., 2022). At the same time, less availability of raw materials and high homogeneity in particle size of the final product may affect its quality (Turunc et al., 2017; Zhang et al., 2020). Both *in vitro* and *in planta* results showed significant inhibition of the *F. oxysporum* which may be attributed to capping of AgNPs by plant secondary constituents that increased its antifungal potential (Mashwani et al., 2016; Marslin et al., 2018; Vanti et al., 2019; Leon-Buitimea et al., 2020) (8, 52, 60,and 74). Our findings regarding the synthesis and antifungal activity suggested that green AgNPs as a novel drug can be used on large industrial scale in order to control the growth of *F. oxysporum*.

The antimicrobial mechanism of metal nanoparticles against plant pathogens has been excellently reviewed (Ali et al., 2020). Although the exact mechanism of action of nanoparticles against microbes is not clear, various mechanisms are thought to be involved. The antimicrobial activities of AgNPs could be the result of a loss of replication activity that inactivates the cellular proteins and enzymes of the pathogens (Feng et al., 2000; Yamanaka et al., 2005). Previously, AgNPs have shown to arrest mycelial growth of *Fusarium oxysporum* (Akpinar et al., 2021), *Fusarium graminearum* (Jian et al., 2022) and *Phytophthora spp.* (Ali et al., 2015). A recent study on the molecular level inhibition of *Fusarium graminearum* in response to AgNPs has shown to induce the expression of azole-related ATP-binding cassette (ABC) transporters and generation of reactive oxygen species, and thus compromise the development, cell structure, cellular energy utilization, and metabolic pathways of this fungus (Jian et al., 2022). Other studies show that nanoparticles penetrate the cell wall and cell membrane and disrupt the cell integrity (Mikhailova, 2020). Some reports suggest nanoparticles induced damage to DNA RNA and proteins, leakage of cellular contents and ultimately death of cells (Kumari et al., 2019; Zhou et al., 2021). Moreover, the application of AgNPs on tomato seedlings has demonstrated to stimulate the antioxidant potential in hydroponics (Noori et al., 2020) which could be considered to enhance the antimicrobial action of AgNPs.

## Conclusions

In the present study, we found *P. geminiflorum* an excellent biological substrate for AgNPs synthesis, most probably due to its rich medicinal phytochemical profile. The plant species showed major medicinal secondary metabolites in qualitative and quantitative analysis. Moreover, the antioxidant activity of the leaf extract was found to be linked with medicinally important secondary metabolites. The AgNPs (100 μg/ml) and the AgNPs-PE (100 μg/ml) inhibited the growth of *F. oxysporum* substantially in the *in vitro* experiment. Further, the *in planta* application of AgNPs alone and combined with plant extract prevented the wilting disease of tomato caused by *F. oxysporum*. Therefore, the antifungal silver nanoparticles synthesized in the current study could be effectively used against *F. oxysporum* as alternatives to hazardous synthetic pesticides. However, further studies are needed to evaluate the antifungal potency of AgNPs alone and in combination with *P. geminiflorum* in the field conditions.

## Data Availability

The original contributions presented in the study are included in the article/Supplementary Material, further inquiries can be directed to the corresponding authors.
